# Supplementary material for: Leveraging electronic health records from two hospital systems identifies male infertility-associated comorbidities across time
Source: Commun Med (Lond). 2025 Sep 1;5:380. doi: 10.1038/s43856-025-01071-7 (PMC12402078; doi:10.1038/s43856-025-01071-7)
Supplement: Supplementary file 2 — Supplementary Information [file 43856_2025_1071_MOESM2_ESM.pdf]

Supplementary Information

Leveraging Electronic Health Records from Two Hospital Systems Identifies Male Infertility Associated Comorbidities Across Time

Sarah Woldemariam[1], Feng Xie[2,3,4,5], Alennie Roldan[1], Jacquelyn Roger[1], Alice S. Tang[1], Tomiko T. Oskotsky[1,6], David K. Stevenson[3], Ruth B. Lathi[7], Aleksandar Rajkovic[8,9,10], Isabel E. Allen[11], Nima Aghaeepour[2,3,4], Michael Eisenberg*[12], Marina Sirota**[1]

*corresponding author; eisenberg@stanford.edu
**corresponding author; Marina.Sirota@ucsf.edu

[1]Bakar Computational Health Sciences Institute, University of California San Francisco, San Francisco, California, USA
[2]Department of Anesthesiology, Perioperative, and Pain Medicine, Stanford University, Stanford, California, USA
[3]Department of Pediatrics, Stanford University, Stanford, California, USA
[4]Department of Biomedical Data Science, Stanford University, Stanford, California, USA
[5]Division of Computational Health Sciences, Department of Surgery, University of Minnesota, Minneapolis, Minnesota, USA
[6]Division of Clinical Informatics and Digital Transformation, Medicine, University of California San Francisco, San Francisco, California, USA
[7]Department of Obstetrics and Gynecology, Stanford University, Stanford, California, USA
[8]Department of Pathology, University of California San Francisco, San Francisco, California, USA
[9]Institute of Human Genetics, University of California San Francisco, San Francisco, California, USA
[10]Department of Obstetrics, Gynecology, and Reproductive Sciences, University of California San Francisco, San Francisco, California, USA
[11]Department of Epidemiology and Biostatistics, University of California San Francisco, San Francisco, California, USA
[12]Department of Urology, Stanford University, Stanford, California, USA

Supplementary Figure 1: Criteria for patient selection at UC.

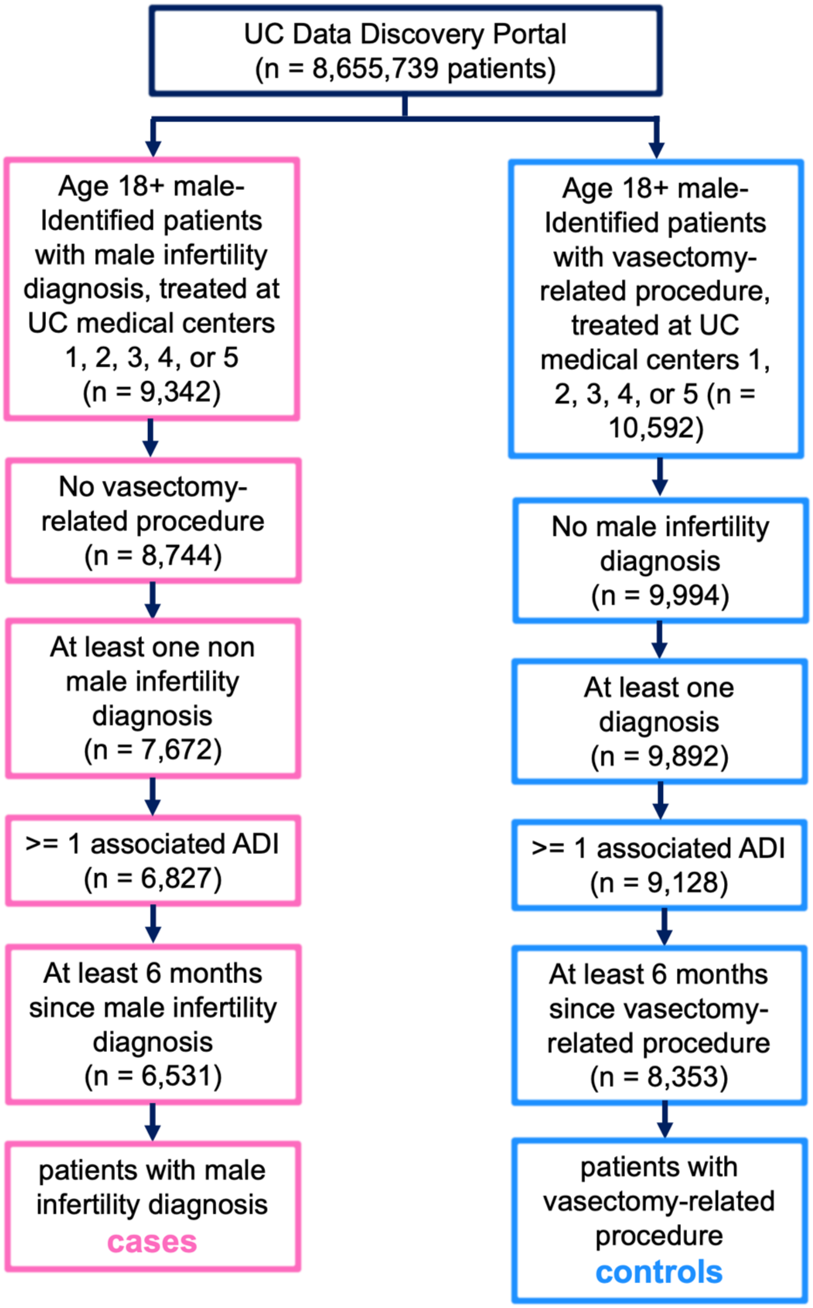

Overview of male infertility (case) and vasectomy (control) patient identification. Pink = male infertility patients (n=6,531 patients); blue = vasectomy patients (n=8,353 patients); ADI = Area Deprivation Index; UC = University of California.

Supplementary Figure 2: Criteria for patient selection at Stanford.

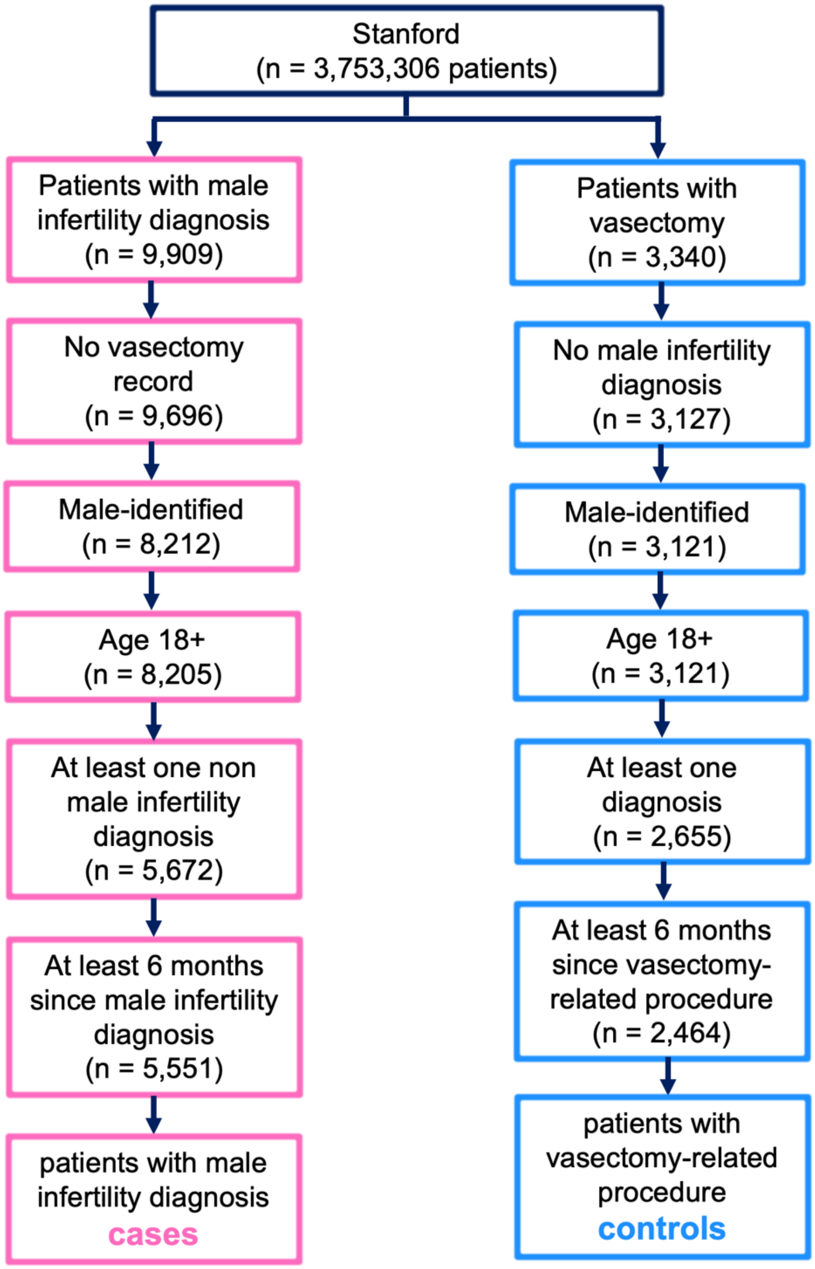

Overview of male infertility (case) and vasectomy (control) patient identification. Pink = male infertility patients (n=5,551 patients); blue = vasectomy patients (n=2,464 patients).

Supplementary Figure 3: Low-dimensional embedding of patients' diagnosis profiles reveals some separation based on patients' male infertility status for diagnoses that were first obtained at any time at UC and Stanford.

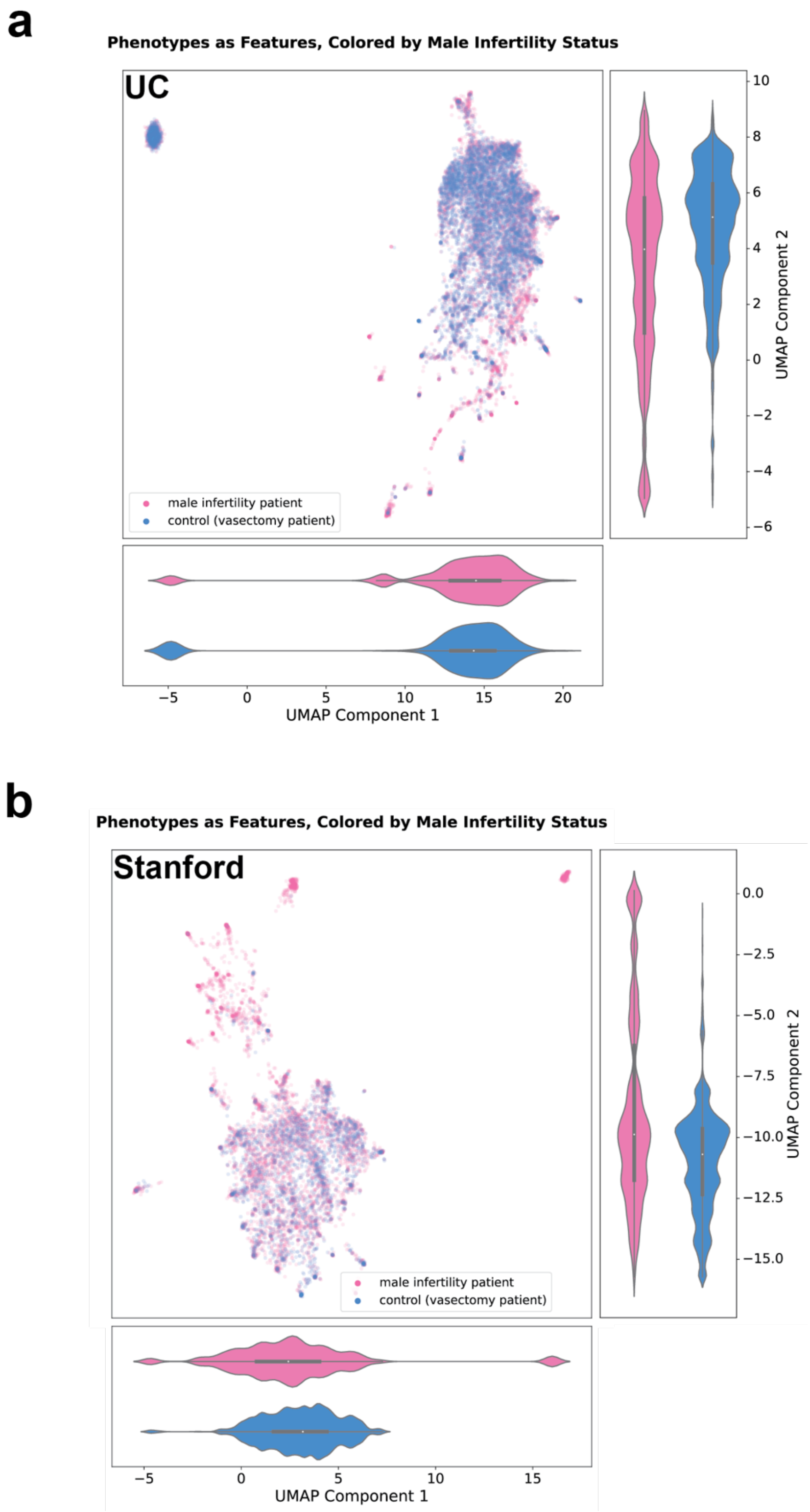

a UMAP of patients' diagnosis profiles of diagnoses first obtained at any time at UC, colored by male infertility status (n=14,884 patients; 1,664 diagnoses as features). **b** UMAP of patients' diagnosis profiles of diagnoses first obtained at any time at Stanford, colored by male infertility status (n=8,015 patients; 1,574 diagnoses as features). For both panels, the bottom violin plot shows distribution of UMAP component 1 based on male infertility status; the right violin plot shows distribution of UMAP component 2 based on male infertility status. Mann-Whitney *U* tests assessed whether the UMAP components significantly differed based on male infertility status, defined as having a significance threshold p-value < 0.05. Pink = patients with male infertility; blue = patients with a vasectomy-related record; UMAP = Uniform Manifold Approximation and Projection; UC = University of California.

Supplementary Figure 4: Low-dimensional embeddings of patients' diagnosis profiles at UC show some separation based on estimated age, location, self- or provider- identified race, self- or provider- identified ethnicity, and area deprivation index.

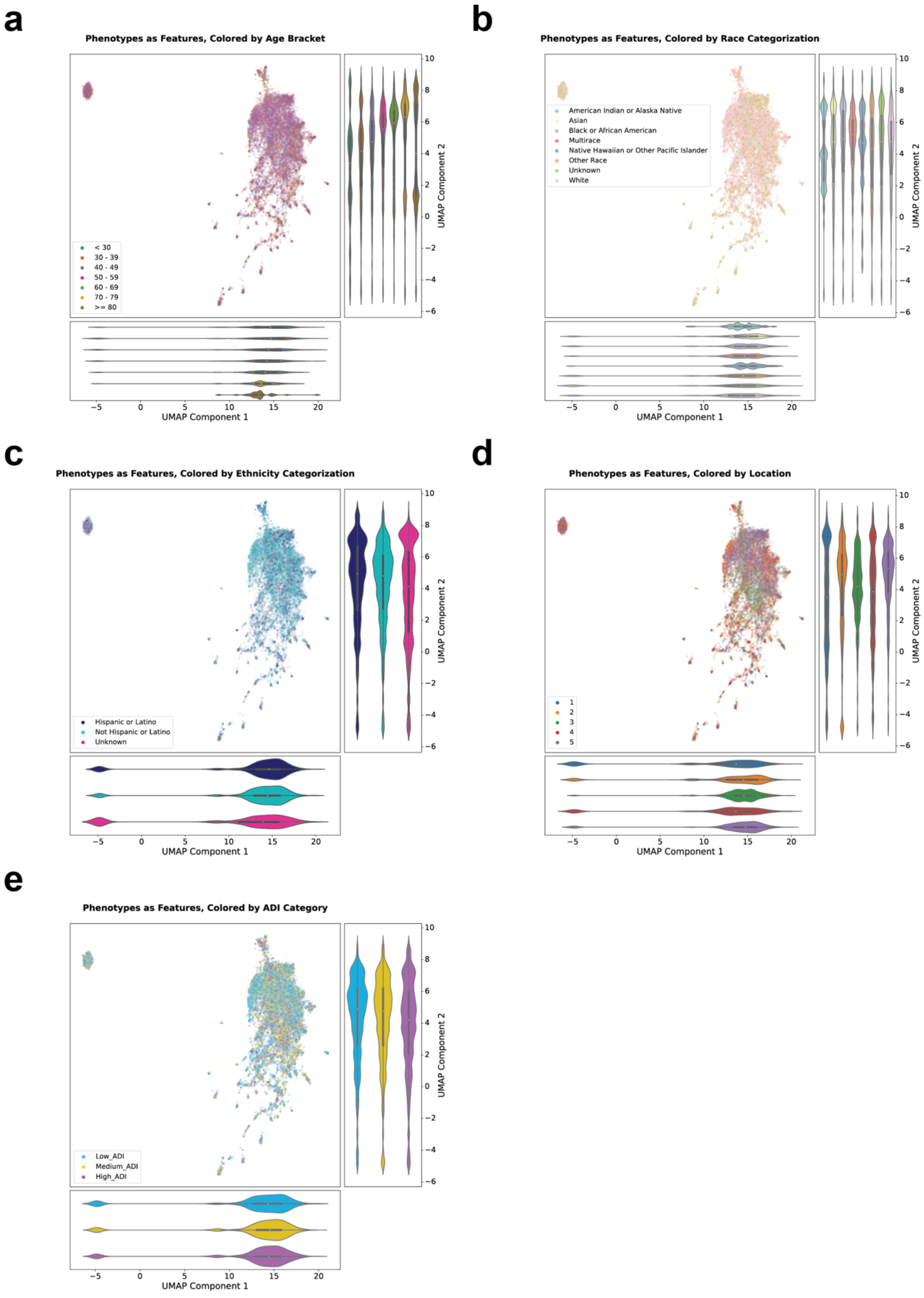

a UMAP of patients' diagnosis profiles of diagnoses that were first obtained at any time, colored by estimated age category. Bottom violin plot shows distribution of UMAP component 1 based on age; the right violin plot shows distribution of UMAP component 2 based on age. < 30 (dark green dots) = estimated age less than 30 years old; 30 - 39 (orange dots) = estimated age between 30 and 39 years old; 40 - 49 (purple dots) = estimated age between 40 and 49 years old; 50 - 59 (pink dots) = estimated age between 50 and 59 years old; 60 - 69 (green dots) = estimated age between 60 and 69 years old; 70 -79 (gold dots) = estimated age between 70 and 79 years old; >= 80 (brown dots) = estimated age aged 80 and above. **b** UMAP of patients' diagnosis profiles of diagnoses that were first obtained at any time, colored by self- or provider-identified race categories. Bottom violin plot shows distribution of UMAP component 1 based on identified race; the right violin plot shows distribution of UMAP component 2 based on identified race. Teal dots = patients identified as American Indian or Alaska Native; yellow dots = patients identified as Asian; purple dots = patients identified as Black or African American; red dots = patients identified as Multirace; blue dots = patients identified as Native Hawaiian or Other Pacific Islander; orange dots = patients identified as Other Race; green dots = patients with an Unknown identified race; pink dots = patients identified as White. **c** UMAP of patients' diagnosis profiles of diagnoses that were first obtained at any time, colored by self- or provider- identified ethnicity categories. Bottom violin plot shows distribution of UMAP component 1 based on identified ethnicity; right violin plot shows distribution of UMAP component 2 based on identified ethnicity. Purple dots = patients identified as Hispanic or Latino; blue dots = patients identified as Not Hispanic or Latino; pink dots = patients with an Unknown identified ethnicity. **d** UMAP of patients' diagnosis profiles of diagnoses that were first obtained at any time, colored by UC location of care. Bottom violin plot shows distribution of UMAP component 1 based on UC location; the right violin plot shows distribution of UMAP component 2 based on UC location. 1 (blue dots) = University of California Medical Center 1; 2 (orange dots) = University of California Medical Center 2; 3 (green dots) = University of California Medical Center 3; 4 (red dots) = University of California Medical Center 4; 5 (purple dots) = University of California Medical Center 5. **e** UMAP of patients' diagnosis profiles of diagnoses that were first obtained any time, colored by area deprivation index categories. Bottom violin plot shows distribution of UMAP component 1 based on area deprivation index category; right violin plot shows distribution of UMAP component 2 based on area deprivation index category. Low_ADI (blue dots) = Patients living in low area deprivation index neighborhoods, where the area deprivation index ranges from 1 to 3; Medium_ADI (yellow dots) = Patients living in medium area deprivation index neighborhoods, where the area deprivation index ranges from 4 to 6; High_ADI (purple dots) = Patients living in high area deprivation index neighborhoods, where the area deprivation index ranges from 7 to 10. For all panels, n=14,884 patients; 1,664 diagnoses as features. Additionally, for all panels, Kruskal-Wallis tests, followed by two-sided Dunn's tests, assessed whether UMAP components significantly differed. Pairwise comparisons were considered significant if they had a Bonferroni-corrected p-value < 0.05. UMAP = Uniform Manifold Approximation and Projection; UC = University of California.

Supplementary Figure 5: Low-dimensional embeddings of patients' diagnosis profiles at Stanford show some separation based on estimated age, self- or provider- identified race, and self- or provider- identified ethnicity.

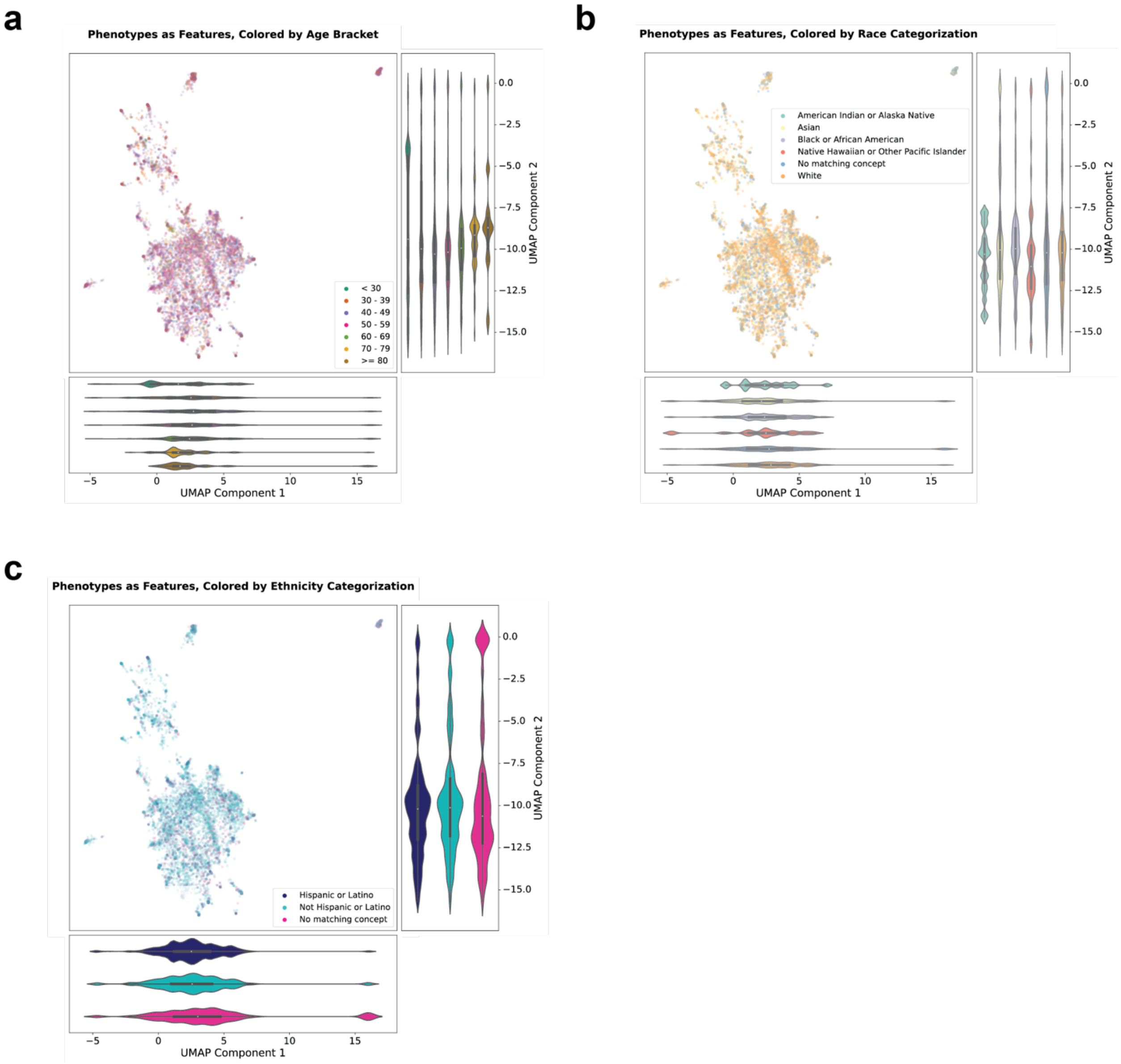

a UMAP of patients' diagnosis profiles of diagnoses that were first obtained at any time, colored by estimated age category. Bottom violin plot shows distribution of UMAP component 1 based on age; the right violin plot shows distribution of UMAP component 2 based on age. < 30 (dark green dots) = estimated age less than 30 years old; 30 - 39 (orange dots) = estimated age between 30 and 39 years old; 40 - 49 (purple dots) = estimated age between 40 and 49 years old; 50 - 59 (pink dots) = estimated age between 50 and 59 years old; 60 - 69 (green dots) = estimated age between 60 and 69 years old; 70 -79 (gold dots) = estimated age between 70

and 79 years old; >= 80 (brown dots) = estimated age aged 80 and above. **b** UMAP of patients' diagnosis profiles of diagnoses that were first obtained at any time, colored by self- or provider-identified race categories. Bottom violin plot shows distribution of UMAP component 1 based on identified race; the right violin plot shows distribution of UMAP component 2 based on identified race. Teal dots = patients identified as American Indian or Alaska Native; yellow dots = patients identified as Asian; purple dots = patients identified as Black or African American; red dots = patients identified as Native Hawaiian or Other Pacific Islander; blue dots = patients identified as belonging to a No matching concept race category; orange dots = patients identified as White. **c** UMAP of patients' diagnosis profiles of diagnoses that were first obtained at any time, colored by self- or provider- identified ethnicity categories. Bottom violin plot shows distribution of UMAP component 1 based on identified ethnicity; right violin plot shows distribution of UMAP component 2 based on identified ethnicity. Purple dots = patients identified as Hispanic or Latino; blue dots = patients identified as Not Hispanic or Latino; pink dots = patients identified as belonging to a No matching concept ethnicity category. For all panels, n=8,015 patients; 1,574 diagnoses as features. Additionally, for all panels, Kruskal-Wallis tests, followed by two-sided Dunn's tests, assessed whether UMAP components significantly differed. Pairwise comparisons were considered significant if they had a Bonferroni-corrected p-value < 0.05. UMAP = Uniform Manifold Approximation and Projection.

Supplementary Figure 6: Low-dimensional embeddings of patients' diagnosis profiles show some separation based on hospital utilization measures before the 6-month cutoff at UC and Stanford.

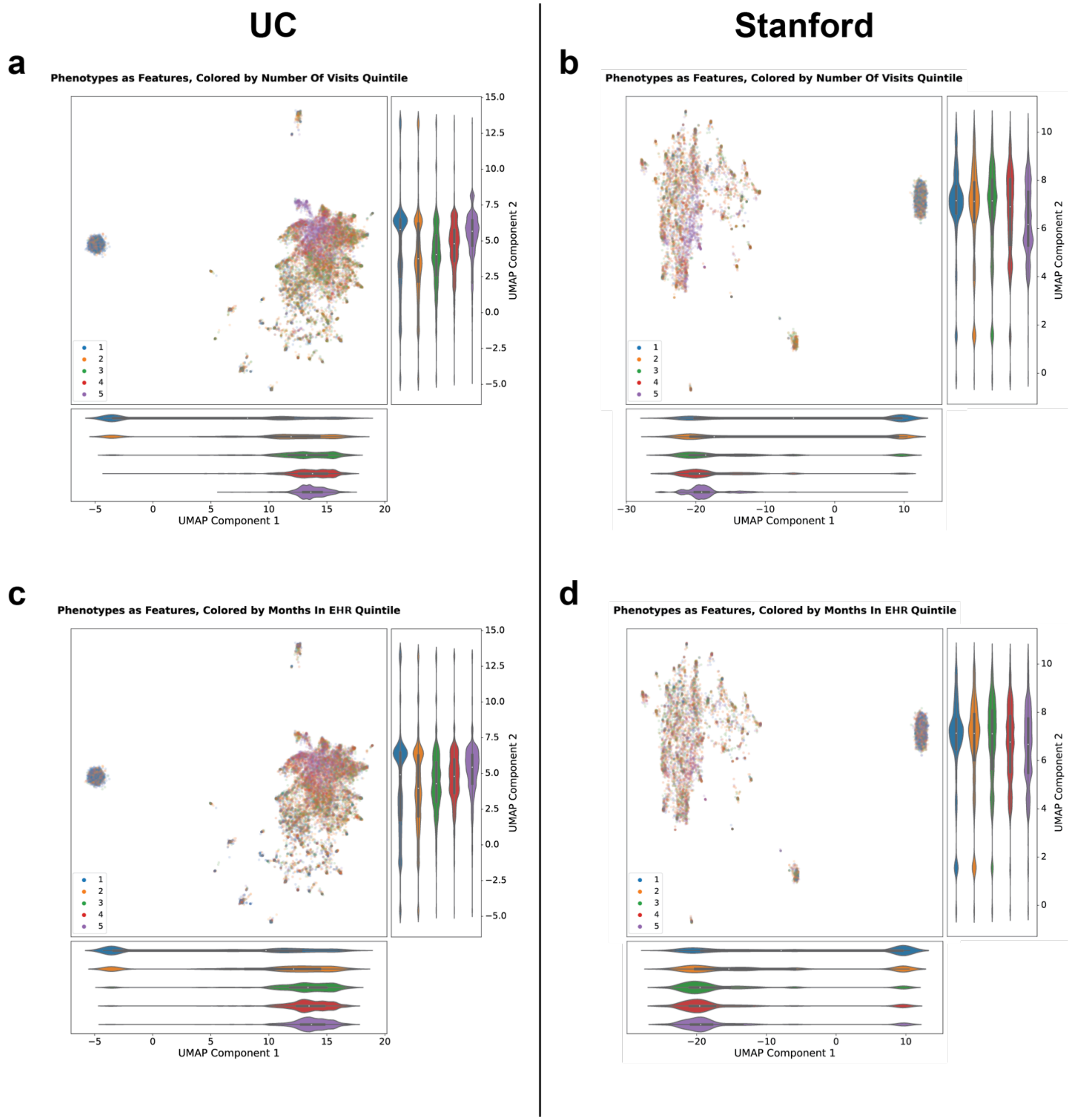

a UMAP of patients' diagnosis profiles of diagnoses that were first obtained before the 6-month cutoff at UC, colored by quintiles of number of visits. Bottom violin plot shows distribution of UMAP component 1 based on quintiles of number of visits; right violin plot shows distribution of UMAP component 2 based on quintiles of number of visits. 1 (blue dots) = quintile 1 (0 - 5

visits); 2 (orange dots) = quintile 2 (6 - 9 visits); 3 (green dots) = quintile 3 (10 - 17 visits); 4 (red dots) = quintile 4 (18 - 33 visits); 5 (purple dots) = quintile 5 (34 - 854 visits). **b** UMAP of patients' diagnosis profiles of diagnoses that were first obtained before the 6-month cutoff at Stanford, colored by quintiles of number of visits. Bottom violin plot shows distribution of UMAP component 1 based on quintiles of number of visits; right violin plot shows distribution of UMAP component 2 based on quintiles of number of visits. 1 (blue dots) = quintile 1 (0 - 6 visits); 2 (orange dots) = quintile 2 (7 - 12 visits); 3 (green dots) = quintile 3 (13 - 21 visits); 4 (red dots) = quintile 4 (22 - 43 visits); 5 (purple dots) = quintile 5 (44 - 1,200 visits). **c** UMAP of patients' diagnosis profiles of diagnoses that were first obtained before the 6-month cutoff, colored by quintiles of months at UC. Bottom violin plot shows distribution of UMAP component 1 based on quintiles of months in the EHR; right violin plot shows distribution of UMAP component 2 based on quintiles of months in the EHR. 1 (blue dots) = quintile 1 (0 - 4 months); 2 (orange dots) = quintile 2 (5 - 10 months); 3 (green dots) = quintile 3 (11 - 29 months); 4 (red dots) = quintile 4 (30 - 58 months); 5 (purple dots) = quintile 5 (59 to 136 months). **d** UMAP of patients' diagnosis profiles of diagnoses that were first obtained before the 6-month cutoff, colored by quintiles of months in the EHR at Stanford. Bottom violin plot shows distribution of UMAP component 1 based on quintiles of months in the EHR; right violin plot shows distribution of UMAP component 2 based on quintiles of months in the EHR. 1 (blue dots) = quintile 1 (0 - 4 months); 2 (orange dots) = quintile 2 (5 - 11 months); 3 (green dots) = quintile 3 (12 - 35 months); 4 (red dots) = quintile 4 (36 - 82 months in the EHR); 5 (purple dots) = quintile 5 (83 - 275 months). For panels **a** and **c** (UC), n=14,812 patients; 1,614 diagnoses as features. For panels **b** and **d** (Stanford), n=7,723 patients; 1,493 diagnoses as features. Additionally, for all panels, Kruskal-Wallis tests, followed by two-sided Dunn's tests, assessed whether UMAP components significantly differed. Pairwise comparisons were considered significant if they had a Bonferroni-corrected $p\text{-value} < 0.05$. UMAP = Uniform Manifold Approximation and Projection; UC = University of California.

Supplementary Figure 7: Low-dimensional embeddings of patients' diagnosis profiles show some separation based on hospital utilization measures after the 6-month cutoff at UC and Stanford.

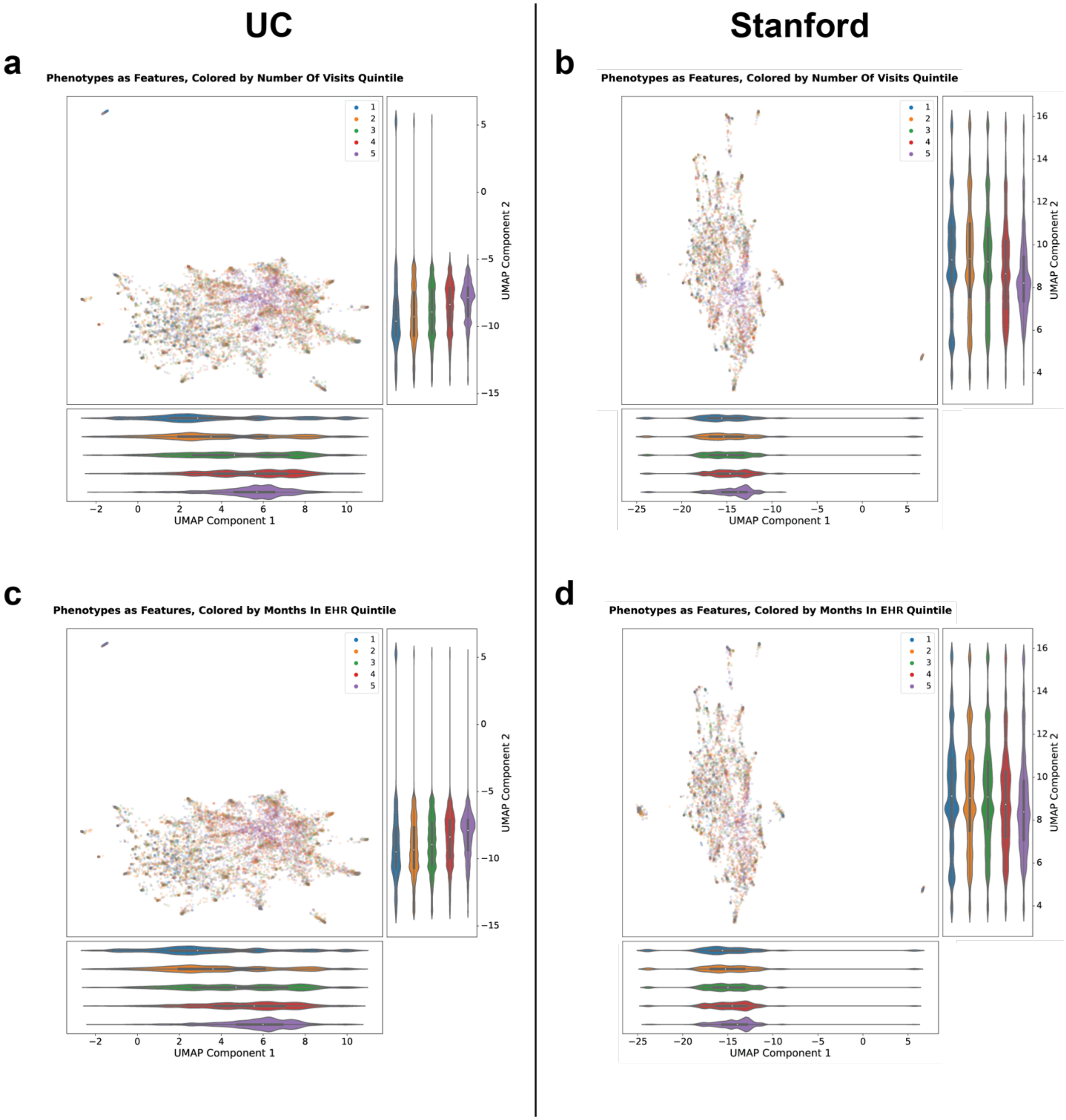

a UMAP of patients' diagnosis profiles of diagnoses that were first obtained after the 6-month cutoff at UC, colored by quintiles of number of visits. Bottom violin plot shows distribution of UMAP component 1 based on quintiles of number of visits; right violin plot shows distribution of UMAP component 2 based on quintiles of number of visits. 1 (blue dots) = quintile 1 (0 - 4

visits); 2 (orange dots) = quintile 2 (5 - 10 visits); 3 (green dots) = quintile 3 (11 - 21 visits); 4 (red dots) = quintile 4 (22 - 43 visits); 5 (purple dots) = quintile 5 (44 to 887 visits). **b** UMAP of patients' diagnosis profiles of diagnoses that were first obtained after the 6-month cutoff at Stanford, colored by quintiles of number of visits. Bottom violin plot shows distribution of UMAP component 1 based on quintiles of number of visits; right violin plot shows distribution of UMAP component 2 based on quintiles of number of visits. 1 (blue dots) = quintile 1 (0 - 8 visits); 2 (orange dots) = quintile 2 (9 - 18 visits); 3 (green dots) = quintile 3 (19 - 34 visits); 4 (red dots) = quintile 4 (35 - 73 visits); 5 (purple dots) = quintile 5 (74 - 1,446 visits). **c** UMAP of patients' diagnosis profiles of diagnoses that were first obtained after the 6-month cutoff, colored by quintiles of months in the EHR at UC. Bottom violin plot shows distribution of UMAP component 1 based on quintiles of months in the EHR; right violin plot shows distribution of UMAP component 2 based on quintiles of months in the EHR. 1 (blue dots) = quintile 1 (0 - 5 months); 2 (orange dots) = quintile 2 (6 - 19 months); 3 (green dots) = quintile 3 (20 - 36 months); 4 (red dots) = quintile 4 (37 - 61 months); 5 (purple dots) = quintile 5 (62 to 129 months). **d** UMAP of patients' diagnosis profiles of diagnoses that were first obtained after the 6-month cutoff, colored by quintiles of months in the EHR at Stanford. Bottom violin plot shows distribution of UMAP component 1 based on quintiles of months in the EHR; right violin plot shows distribution of UMAP component 2 based on quintiles of months in the EHR. 1 (blue dots) = quintile 1 (0 - 12 months); 2 (orange dots) = quintile 2 (13 - 32 months); 3 (green dots) = quintile 3 (33 - 60 months); 4 (red dots) = quintile 4 (61 - 99 months); 5 (purple dots) = quintile 5 (100 to 231 months). For panels **a** and **c** (UC), n=9,234 patients; 1,562 diagnoses as features. For panels **b** and **d** (Stanford), n=5,564 patients; 1,452 diagnoses as features. Additionally, for all panels, Kruskal-Wallis tests, followed by two-sided Dunn's tests, assessed whether UMAP components significantly differed. Pairwise comparisons were considered significant if they had a Bonferroni-corrected p-value < 0.05. UMAP = Uniform Manifold Approximation and Projection; UC = University of California.

Supplementary Figure 8: Ln-ln plots show that the odds ratios of diagnoses from primary analyses correlate with the odds ratios of diagnosis from sensitivity analyses at both UC and Stanford before the 6-month cutoff.

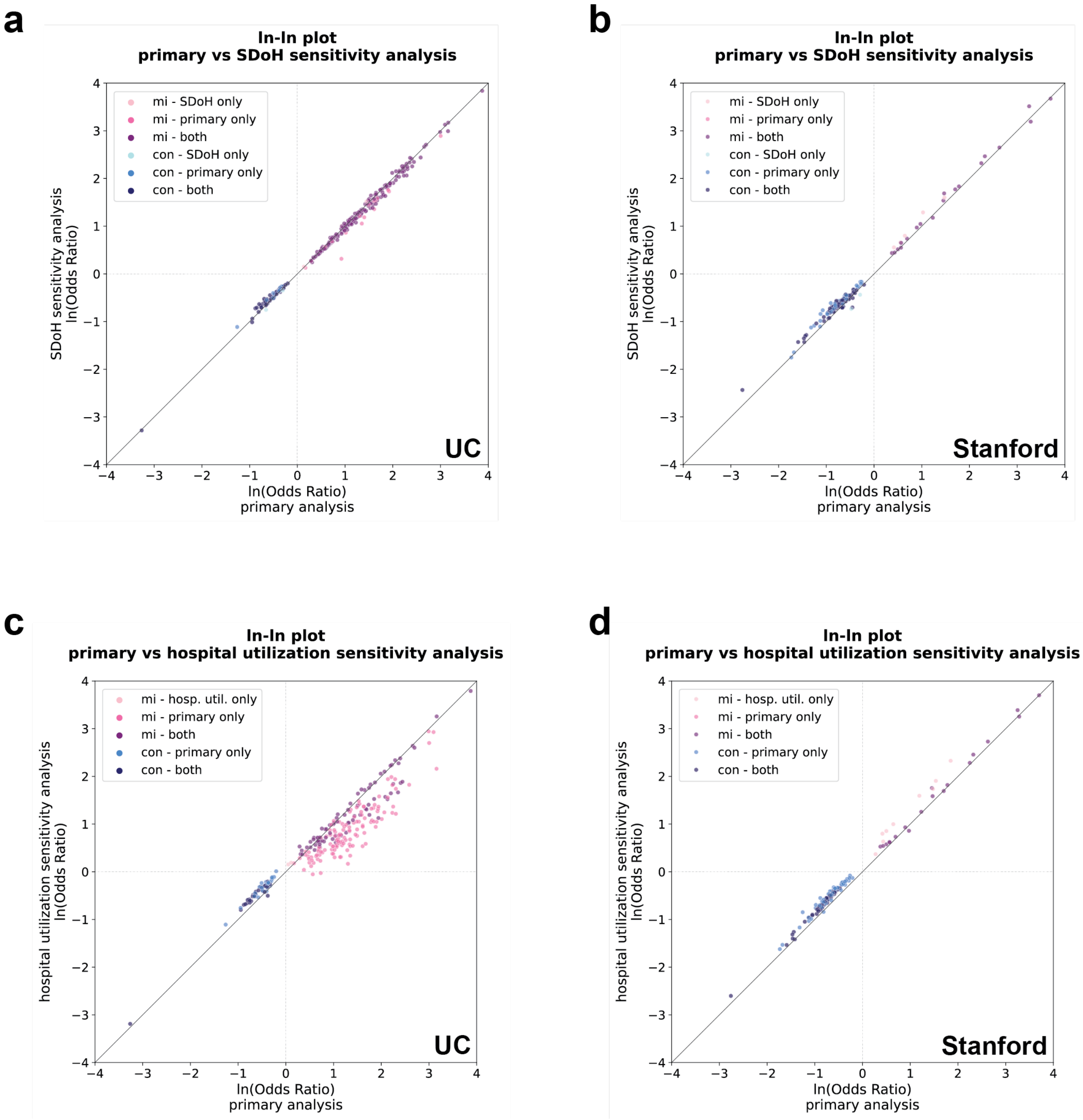

a UC ln-ln plot of the ln(odds ratio) of diagnoses in the social determinants of health (SDoH) sensitivity analysis that were first obtained before the 6-month cutoff, versus the ln(odds ratio) of diagnoses in the primary analysis that were first obtained before the 6-month cutoff. **b** Stanford ln-ln plot of the ln(odds ratio) of diagnoses in the SDoH sensitivity analysis that were first obtained before the 6-month cutoff, versus the ln(odds ratio) of diagnoses in the primary

analysis that were first obtained before the 6-month cutoff. **c** UC ln-ln plot of the ln(odds ratio) of diagnoses in the hospital utilization sensitivity analysis for diagnoses first obtained before the 6-month cutoff, versus the ln(odds ratio) of diagnoses in the primary analysis that were first obtained before the 6-month cutoff. **d** Stanford ln-ln plot of the ln(odds ratio) of diagnoses in the hospital utilization sensitivity analysis for diagnoses first obtained before the 6-month cutoff, versus the ln(odds ratio) of diagnoses in the primary analysis that were first obtained before the 6-month cutoff. Pearson correlation coefficient was used to assess correlation and significance. For panels **a** and **b**, mi - SDoH only (light pink dots) = diagnoses that are positively associated with male infertility in the SDoH sensitivity analysis only; mi - primary only (pink dots) = diagnoses that are positively associated with male infertility in the primary analysis only; mi – both (dark pink dots) = diagnoses that are positively associated in both the primary analysis and the SDoH sensitivity analysis; con - SDoH only (light blue dots) = diagnoses that are negatively associated with male infertility in the SDoH sensitivity analysis only; con - primary only (blue dots) = diagnoses that are negatively associated with male infertility in the primary analysis only; con – both (dark blue dots) = diagnoses that are negatively associated in both the primary analysis and the SDoH sensitivity analysis. For panels **c** and **d**, mi - hosp. util. only (light pink dots) = diagnoses that are positively associated with male infertility in the hospital utilization sensitivity analysis only; mi - primary only (pink dots) = diagnoses that are positively associated with male infertility in the primary analysis only; mi – both (dark pink dots) = diagnoses that are positively associated in both the primary analysis and the hospital utilization sensitivity analysis; con - primary only (blue dots) = diagnoses that are negatively associated with male infertility in the primary analysis only; con – both (dark blue dots) = diagnoses that are negatively associated in both the primary analysis and the hospital utilization sensitivity analysis. n=6,531 UC patients with male infertility; n=8,353 UC patients with vasectomy-related record; n=5,551 Stanford patients with male infertility, n=2,464 Stanford patients with vasectomy-related record. UC = University of California.

Supplementary Figure 9: A number of diagnoses are negatively associated with male infertility across UC and Stanford before the 6-month cutoff.

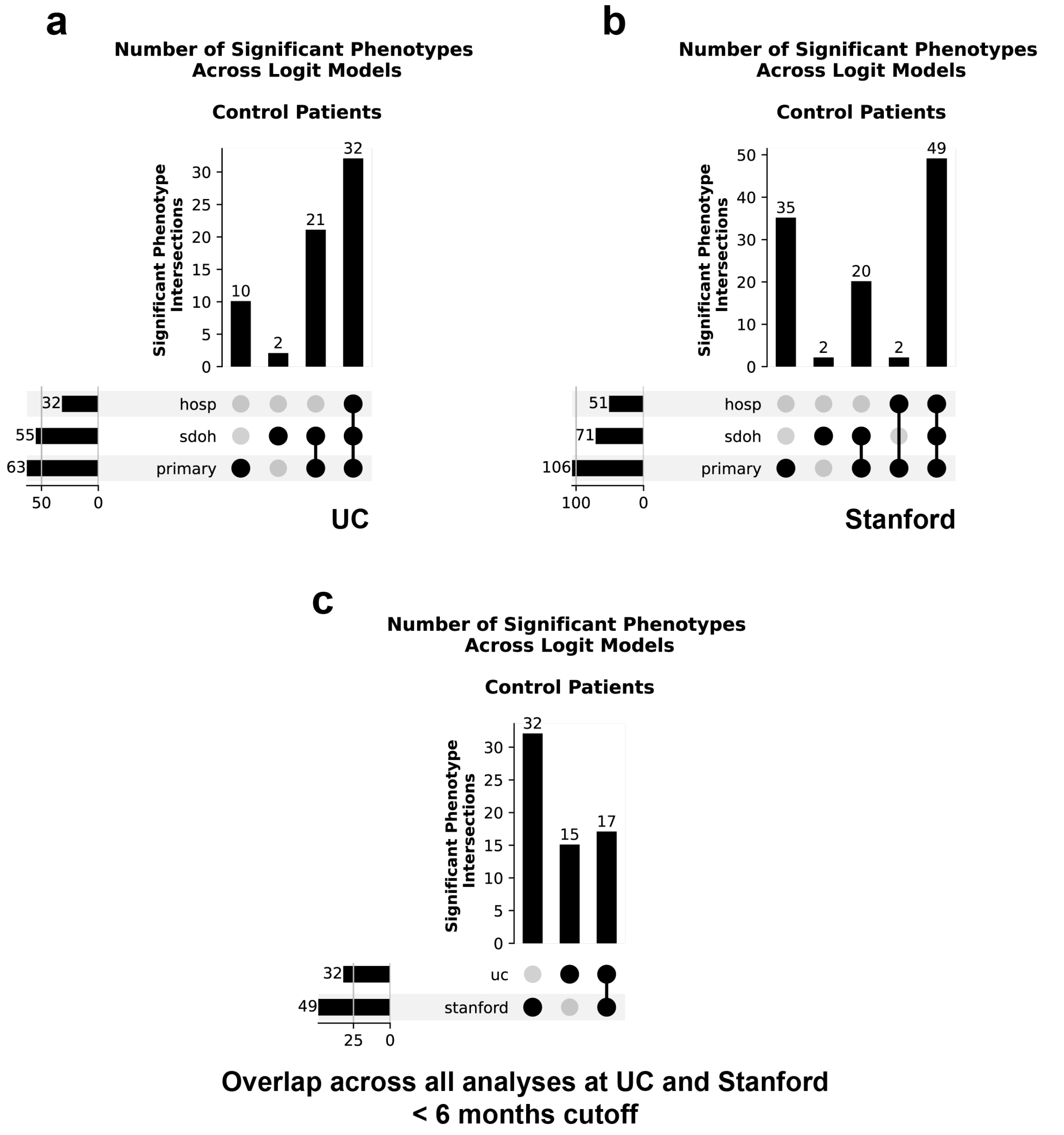

a Upset plot of shared significant diagnoses negatively associated with male infertility across the primary analysis and both sensitivity analyses for diagnoses first obtained before the 6-month cutoff at UC. **b** Upset plot of shared significant diagnoses negatively associated with male infertility across the primary analysis and both sensitivity analyses for diagnoses first obtained

before the 6-month cutoff at Stanford. **c** Upset plot of shared significant diagnoses negatively associated with male infertility across all six analyses for diagnoses first obtained before the 6-month cutoff across UC and Stanford. For all panels, horizontal rows indicate a specific analysis, and bar charts indicate the number of overlapping significant diagnoses for a given combination of analyses. hosp = hospital utilization sensitivity analysis; primary = primary analysis; sdoh = social determinants of health sensitivity analysis; uc = University of California.

Supplementary Figure 10: Ln-ln plots show that the odds ratios of diagnoses from primary analyses correlate with the odds ratios of diagnosis from sensitivity analyses at both UC and Stanford after the 6-month cutoff.

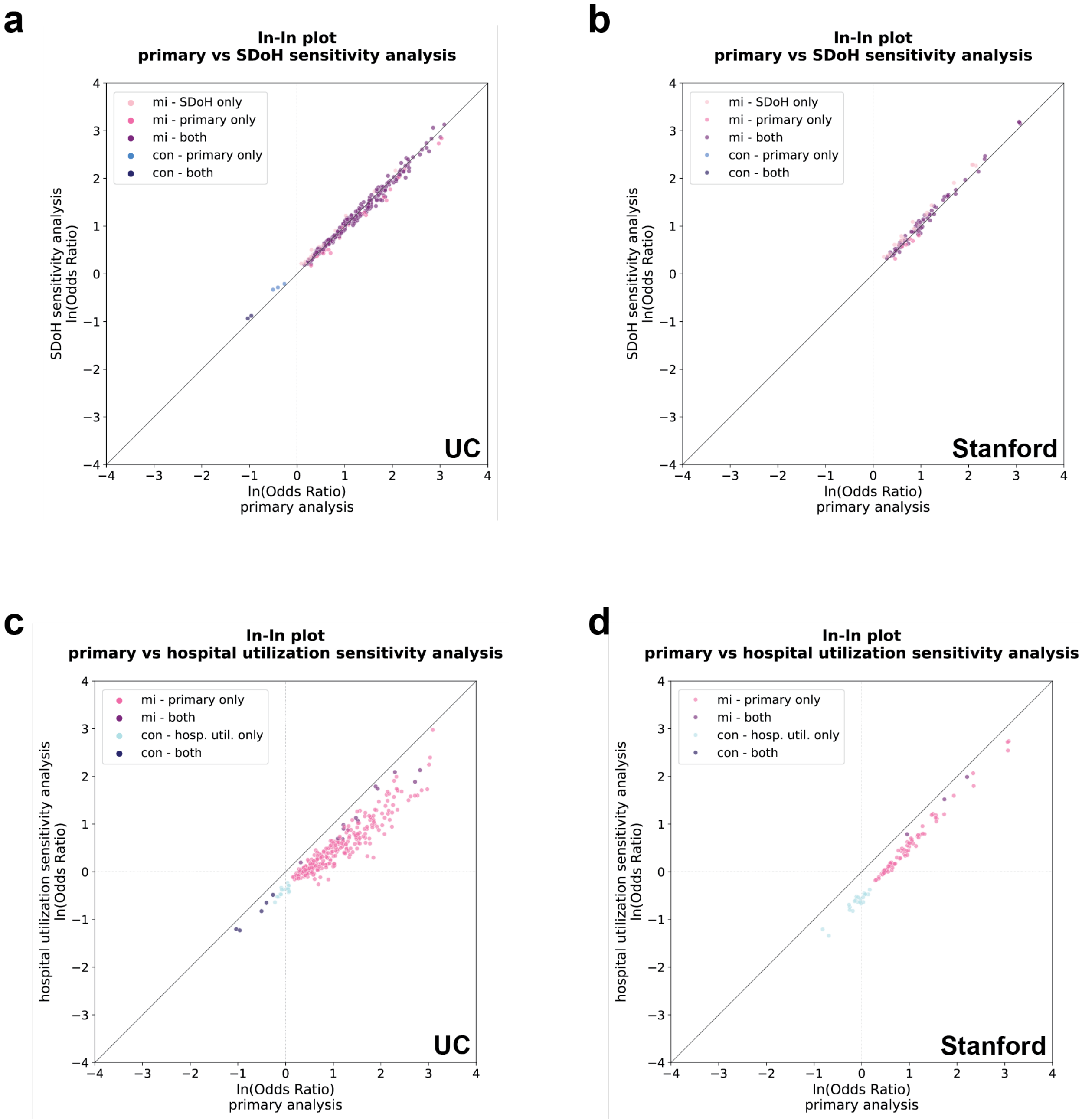

a UC ln-ln plot of the ln(odds ratio) of diagnoses in the social determinants of health (SDoH) sensitivity analysis that were first obtained after the 6-month cutoff, versus the ln(odds ratio) of diagnoses in the primary analysis that were first obtained after the 6-month cutoff. **b** Stanford ln-ln plot of the ln(odds ratio) of diagnoses in the SDoH sensitivity analysis that were first obtained

after the 6-month cutoff, versus the ln(odds ratio) of diagnoses in the primary analysis that were first obtained after the 6-month cutoff. **c** UC ln-ln plot of the ln(odds ratio) of diagnoses in the hospital utilization sensitivity analysis for diagnoses first obtained after the 6-month cutoff, versus the ln(odds ratio) of diagnoses in the primary analysis that were first obtained after the 6-month cutoff. **d** Stanford ln-ln plot of the ln(odds ratio) of diagnoses in the hospital utilization sensitivity analysis for diagnoses first obtained after the 6-month cutoff, versus the ln(odds ratio) of diagnoses in the primary analysis that were first obtained after the 6-month cutoff. Pearson correlation coefficient was used to assess correlation and significance. For panels **a** and **b**, mi - SDoH only (light pink dots) = diagnoses that are positively associated with male infertility in the SDoH sensitivity analysis only; mi - primary only (pink dots) = diagnoses that are positively associated with male infertility in the primary analysis only; mi – both (dark pink dots) = diagnoses that are positively associated in both the primary analysis and the SDoH sensitivity analysis; con - primary only (blue dots) = diagnoses that are negatively associated with male infertility in the primary analysis only; con – both (dark blue dots) = diagnoses that are negatively associated in both the primary analysis and the SDoH sensitivity analysis. For panels **c** and **d**, mi - primary only (pink dots) = diagnoses that are positively associated with male infertility in the primary analysis only; mi – both (dark pink dots) = diagnoses that are positively associated in both the primary analysis and the hospital utilization sensitivity analysis; con - primary only (blue dots) = diagnoses that are negatively associated with male infertility in the primary analysis only; con – both (dark blue dots) = diagnoses that are negatively associated in both the primary analysis and the hospital utilization sensitivity analysis. n=6,531 UC patients with male infertility; n=8,353 UC patients with vasectomy-related record; n=5,551 Stanford patients with male infertility, n=2,464 Stanford patients with vasectomy-related record. UC = University of California.

Supplementary Figure 11: Several diagnoses are negatively associated with male infertility across analyses at UC after the 6-month cutoff.

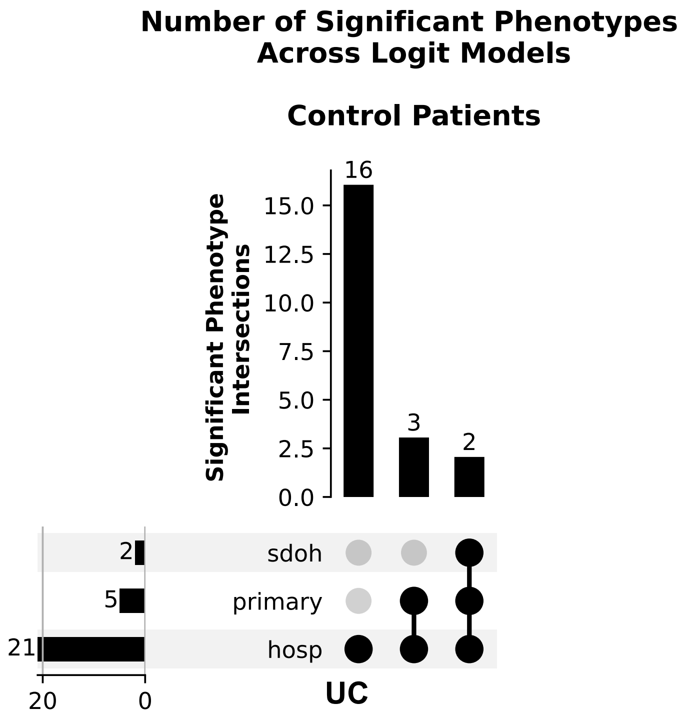

Upset plot of shared significant diagnoses negatively associated with male infertility across the primary analysis and both sensitivity analyses for diagnoses first obtained after the 6-month cutoff at UC. Horizontal rows indicate a specific analysis, and bar charts indicate the number of overlapping significant diagnoses for a given combination of analyses. hosp = hospital utilization sensitivity analysis; primary = primary analysis; sdoh = social determinants of health sensitivity analysis. UC = University of California.

Supplementary Figure 12: Kaplan-Meier visualizations of receiving a given diagnosis based on male infertility status at UC.

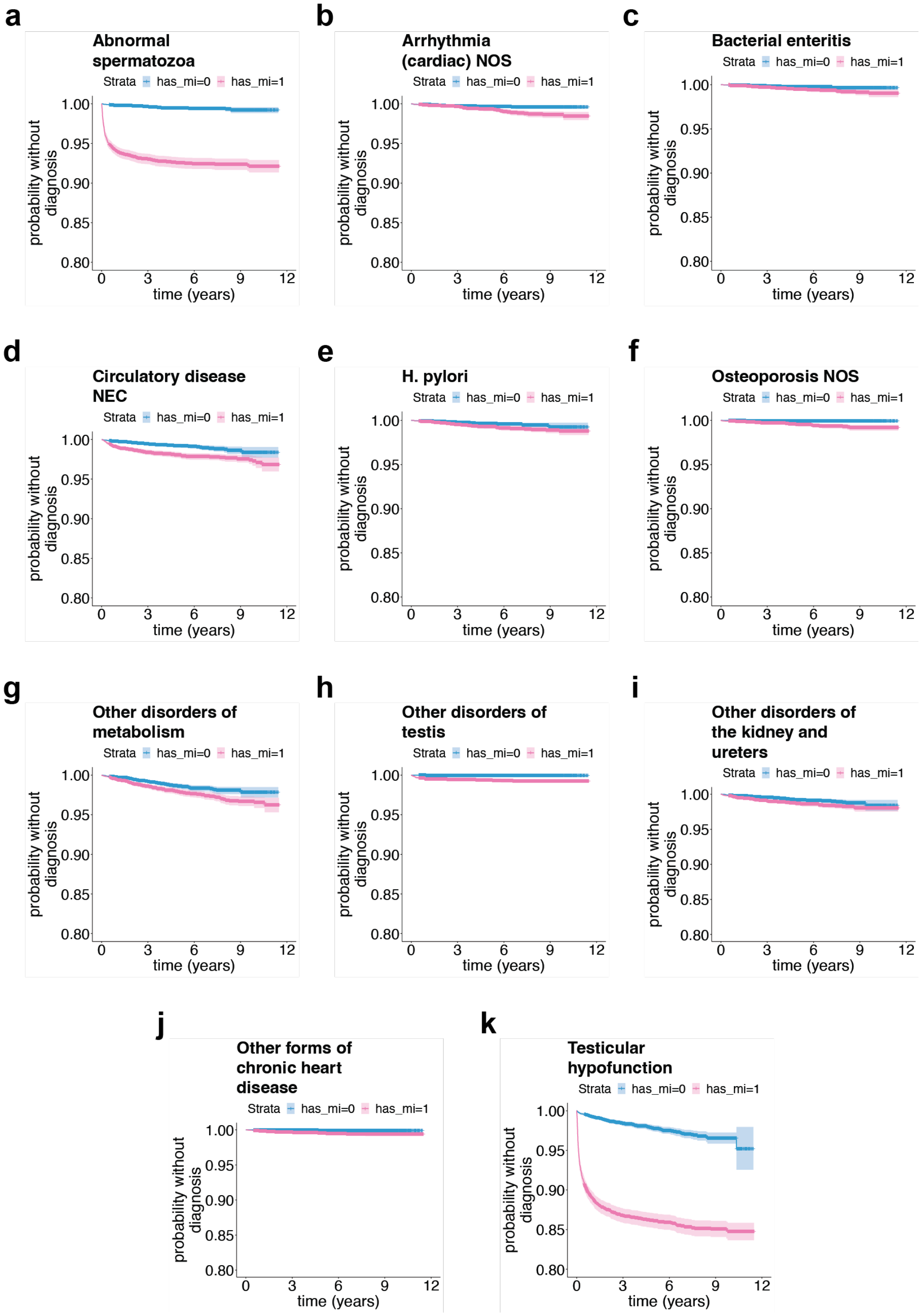

Kaplan-Meier curves for **a** Abnormal spermatozoa, **b** Arrythmia, **c** Bacterial enteritis, **d** Circulatory disease, **e** *H. Pylori*, **f** Osteoporosis, **g** Other disorders of metabolism, **h** Other disorders of testis, **i** Other disorders of the kidney and ureters, **j** Other forms of chronic heart

disease, and **k** Testicular hypofunction. These are the 11 diagnoses male infertility patients at UC have a higher risk of receiving relative to vasectomy patients after their first male infertility diagnosis (for male infertility patients) or vasectomy-related record (for vasectomy patients). has_mi=0 (blue curve) = Kaplan-Meier curve for vasectomy patients, with error bars indicating 95% confidence interval; has_mi=1 (pink curve) = Kaplan-Meier curve for male infertility patients, with error bars indicating 95% confidence interval. For each panel, the top curve corresponds to the blue curve, and the bottom curve corresponds to the pink curve. NOS = not otherwise specified; NES = not elsewhere specified.
