# Supplementary material for: Leveraging electronic health records from two hospital systems identifies male infertility-associated comorbidities across time
Source: Commun Med (Lond). 2025 Sep 1;5:380. doi: 10.1038/s43856-025-01071-7 (PMC12402078; doi:10.1038/s43856-025-01071-7)
Supplement: Supplementary file 3 — Description of Additional Supplementary Files [file 43856_2025_1071_MOESM3_ESM.pdf]

File Name: Supplementary Data 1

Description: Concepts used to identify male infertility and vasectomy patients.

mi_concepts contains concepts used to identify male infertility patients;

vasectomy_concepts contains concepts used to identify vasectomy patients;

legend contains column descriptions.

File Name: Supplementary Data 2

Description: Stanford demographics. n = number of patients; sd = standard deviation; number of visits before = number of visits before the 6-month cutoff; number of visits after = number of visits after the 6-month cutoff; months in EHR before = months in EHR before the 6-month cutoff (calculated as length of time between patient's last visit and patient's first visit before the 6-month cutoff); months in EHR after = months in EHR after the 6-month cutoff (calculated as length of time between patient's last visit and patient's first visit after the 6-month cutoff).

File Name: Supplementary Data 3

Description: Mann-Whitney *U* test and Kruskal-Wallis test results for comparing UMAP components based on features of interest.

uc_mannwhitney sheet contains Mann-Whitney *U* test statistics and p-values comparing UMAP components based on male infertility status at UC;

stanford_mannwhitney sheet contains Mann-Whitney *U* test statistics and p-values comparing UMAP components based on male infertility status at Stanford;

uc_kruskalwallis contains Kruskal-Wallis test statistics and p-values comparing UMAP components based on features of interest at UC;

stanford_kruskalwallis contains Kruskal-Wallis test statistics and p-values comparing UMAP components based on features of interest at Stanford;

legend describes UMAP comparisons made.

File Name: Supplementary Data 4

Description: p-values of post hoc Dunn's tests of pairwise comparisons of UMAP components based on features of interest at UC, represented at covariance matrices.

location_axis1 contains results comparing UMAP component 1 distributions of patients' diagnosis profiles of diagnoses first obtained at anytime, based on UC location;

location_axis2 contains results comparing UMAP component 2 distributions of patients' diagnosis profiles of diagnoses first obtained at anytime, based on UC location;

age_axis1 contains results comparing UMAP component 1 distributions of patients' diagnosis profiles of diagnoses first obtained at anytime, based on age category;
age_axis2 contains results comparing UMAP component 2 distributions of patients' diagnosis profiles of diagnoses first obtained at anytime, based on age category;

race_axis1 contains results comparing UMAP component 1 distributions of patients' diagnosis profiles of diagnoses first obtained at anytime, based on self- or provider- identified race category;

race_axis2 contains results comparing UMAP component 2 distributions of patients' diagnosis profiles of diagnoses first obtained at anytime, based on self- or provider- identified race category;

ethnicity_axis1 contains results comparing UMAP component 1 distributions of patients' diagnosis profiles of diagnoses first obtained at anytime, based on self- or provider- identified ethnicity category;

ethnicity_axis2 contains results comparing UMAP component 2 distributions of patients' diagnosis profiles of diagnoses first obtained at anytime, based on self- or provider- identified ethnicity category;

adi_axis1 contains results comparing UMAP component 1 distributions of patients' diagnosis profiles of diagnoses first obtained at anytime, based on area deprivation index category;

adi_axis2 contains results comparing UMAP component 2 distributions of patients' diagnosis profiles of diagnoses first obtained at anytime, based on area deprivation index category;

visits_before_axis1 contains results comparing UMAP component 1 distributions of patients' diagnosis profiles of diagnoses first obtained before the 6-month cutoff, based on number of visits before the 6-month cutoff;

visits_before_axis2 contains results comparing UMAP component 2 distributions of patients' diagnosis profiles of diagnoses first obtained before the 6-month cutoff, based on number of visits before the 6-month cutoff;

visits_after_axis1 contains results comparing UMAP component 1 distributions of patients' diagnosis profiles of diagnoses first obtained after the 6-month cutoff, based on number of visits after the 6-month cutoff;

visits_after_axis2 contains results comparing UMAP component 2 distributions of patients' diagnosis profiles of diagnoses first obtained after the 6-month cutoff, based on number of visits after the 6-month cutoff;

mths_EHR_before_axis1 contains results comparing UMAP component 1 distributions of patients' diagnosis profiles of diagnoses first obtained before the 6-month cutoff, based on months in the EHR before the 6-month cutoff;

mths_EHR_before_axis2 contains results comparing UMAP component 2 distributions of patients' diagnosis profiles of diagnoses first obtained before the 6-month cutoff, based on months in the EHR before the 6-month cutoff;

mths_EHR_after_axis1 contains results comparing UMAP component 1 distributions of patients' diagnosis profiles of diagnoses first obtained after the 6-month cutoff, based on months in the EHR after the 6-month cutoff;

mths_EHR_after_axis2 contains results comparing UMAP component 2 distributions of patients' diagnosis profiles of diagnoses first obtained after the 6-month cutoff, based on months in the EHR after the 6-month cutoff.

File Name: Supplementary Data 5

Description: p-values of post hoc Dunn's tests of pairwise comparisons of UMAP components based on features of interest at Stanford, represented at covariance matrices.

age_axis1 contains results comparing UMAP component 1 distributions of patients' diagnosis profiles of diagnoses first obtained at anytime, based on age category;

age_axis2 contains results comparing UMAP component 2 distributions of patients' diagnosis profiles of diagnoses first obtained at anytime, based on age category;

race_axis1 contains results comparing UMAP component 1 distributions of patients' diagnosis profiles of diagnoses first obtained at anytime, based on self- or provider- identified race category;

race_axis2 contains results comparing UMAP component 2 distributions of patients' diagnosis profiles of diagnoses first obtained at anytime, based on self- or provider- identified race category;

ethnicity_axis1 contains results comparing UMAP component 1 distributions of patients' diagnosis profiles of diagnoses first obtained at anytime, based on self- or provider- identified ethnicity category;

ethnicity_axis2 contains results comparing UMAP component 2 distributions of patients' diagnosis profiles of diagnoses first obtained at anytime, based on self- or provider- identified ethnicity category;

visits_before_axis1 contains results comparing UMAP component 1 distributions of patients' diagnosis profiles of diagnoses first obtained before the 6-month cutoff, based on number of visits before the 6-month cutoff;

visits_before_axis2 contains results comparing UMAP component 2 distributions of patients' diagnosis profiles of diagnoses first obtained before the 6-month cutoff, based on number of visits before the 6-month cutoff;

visits_after_axis1 contains results comparing UMAP component 1 distributions of patients' diagnosis profiles of diagnoses first obtained after the 6-month cutoff, based on number of visits after the 6-month cutoff;

visits_after_axis2 contains results comparing UMAP component 2 distributions of patients' diagnosis profiles of diagnoses first obtained after the 6-month cutoff, based on number of visits after the 6-month cutoff;

mths_EHR_before_axis1 contains results comparing UMAP component 1 distributions of patients' diagnosis profiles of diagnoses first obtained before the 6-month cutoff, based on months in the EHR before the 6-month cutoff;

mths_EHR_before_axis2 contains results comparing UMAP component 2 distributions of patients' diagnosis profiles of diagnoses first obtained before the 6-month cutoff, based on months in the EHR before the 6-month cutoff;

mths_EHR_after_axis1 contains results comparing UMAP component 1 distributions of patients' diagnosis profiles of diagnoses first obtained after the 6-month cutoff, based on months in the EHR after the 6-month cutoff;

mths_EHR_after_axis2 contains results comparing UMAP component 2 distributions of patients' diagnosis profiles of diagnoses first obtained after the 6-month cutoff, based on months in the EHR after the 6-month cutoff.

File Name: Supplementary Data 6

Description: Comparison of number of diagnoses per patient based on outlier cluster membership at UC and Stanford (see Figure 2a and Figure 2b). Note, patient counts less than or equal to 10 are set to 10.

UC sheet contains Mann-Whitney *U* test results comparing the outlier cluster with the nonoutlier cluster for UC (see Figure2a, which shows the UMAP of patients' diagnosis profiles before the 6-month cutoff).

Stanford sheet contains Mann-Whitney *U* test results comparing the outlier cluster with the nonoutlier cluster for Stanford (see Figure2b, which shows the UMAP of patients' diagnosis profiles before the 6-month cutoff).

legend contains column and variable descriptions.

File Name: Supplementary Data 7

Description: Distribution of time delta in months between first diagnosis time and patients' first male infertility diagnosis or vasectomy-related record. Includes descriptive statistics, including mean, standard deviation, and quartiles. Diagnoses are represented as phecode-corresponding phenotypes.

before_6m_cutoff_UC contains distribution of time delta before the 6-month cutoff for UC patients;

after_6m_cutoff_UC contains distribution of time delta after the 6-month cutoff for UC patients;

before_6m_cutoff_Stanford contains distribution of time delta before the 6-month cutoff for Stanford patients;

after_6m_cutoff_Stanford contains distribution of time delta after the 6-month cutoff for Stanford patients;

legend contains column descriptions.

File Name: Supplementary Data 8

Description: Logistic regression results for each diagnosis first obtained before the 6-month cutoff at UC. Note, patient counts less than or equal to 10 are set to 10.

before_primary contains results for the primary analysis;

before_sdoh contains results for the social determinants of health sensitivity analysis;

before_hosp contains results for the hospital utilization sensitivity analysis;

legend contains column descriptions.

File Name: Supplementary Data 9

Description: Logistic regression results for each diagnosis first obtained before the 6-month cutoff at Stanford. Note, patient counts less than or equal to 10 are set to 10.

before_primary contains results for the primary analysis;

before_sdoh contains results for the social determinants of health sensitivity analysis;

before_hosp contains results for the hospital utilization sensitivity analysis;

legend contains column descriptions.

File Name: Supplementary Data 10

Description: Pearson correlation coefficients of primary analyses versus sensitivity analyses for UC and Stanford, before and after the 6-month cutoff.

File Name: Supplementary Data 11

Description: Overlapping diagnoses that are significantly associated with male infertility across analyses at UC for diagnoses first obtained before the 6-month cutoff. phenotype corresponds to diagnosis; phecode category is the disease category of the phenotype (i.e., diagnosis).

mi_before_hosp contains significant diagnoses positively associated with male infertility in the hospital utilization sensitivity analysis only;

mi_before_primary contains significant diagnoses positively associated with male infertility in the primary analysis only;

mi_before_primary_hosp contains significant diagnoses positively associated with male infertility in the primary analysis and hospital utilization sensitivity analysis;

mi_before_primary_sdoh contains significant diagnoses positively associated with male infertility in the primary analysis and social determinants of health sensitivity analysis;

mi_before_primary_sdoh_hosp contains significant diagnoses positively associated with male infertility in all three analyses (primary, social determinants of health, and hospital utilization);

mi_before_sdoh contains significant diagnoses positively associated with male infertility in the social determinants of health sensitivity analysis only;

mi_before_sdoh_hosp contains significant diagnoses positively associated with male infertility in the social determinants of health and hospital utilization sensitivity analyses;

con_before_primary contains significant diagnoses negatively associated with male infertility in the primary analysis only;

con_before_primary_sdoh contains significant diagnoses negatively associated with male infertility in the primary analysis and social determinants of health sensitivity analysis;

con_before_primary_sdoh_hosp contains significant diagnoses negatively associated with male infertility in all three analyses (primary, social determinants of health, and hospital utilization);

con_before_sdoh contains significant diagnoses negatively associated with male infertility in the social determinants of health sensitivity analysis only.

File Name: Supplementary Data 12

Description: Overlapping diagnoses that are significantly associated with male infertility across analyses at Stanford for diagnoses first obtained before the 6-month cutoff. phenotype corresponds to diagnosis, which are represented by phenotypes; phecode category is the disease category of the phenotype (i.e., diagnosis).

mi_before_hosp contains significant diagnoses positively associated with male infertility in the hospital utilization sensitivity analysis only;

mi_before_primary_sdoh_hosp contains significant diagnoses positively associated with male infertility in all three analyses (primary, social determinants of health, and hospital utilization);

mi_before_sdoh contains significant diagnoses positively associated with male infertility in the social determinants of health sensitivity analysis only;

mi_before_sdoh_hosp contains significant diagnoses positively associated with male infertility in the social determinants of health and hospital utilization sensitivity analyses;

con_before_primary contains significant diagnoses negatively associated with male infertility in the primary analysis only;

con_before_primary_hosp contains significant diagnoses negatively associated with male infertility in the primary analysis and hospital utilization sensitivity analysis;

con_before_primary_sdoh contains significant diagnoses negatively associated with male infertility in the primary analysis and social determinants of health sensitivity analysis;

con_before_primary_sdoh_hosp contains significant diagnoses negatively associated with male infertility in all three analyses (primary, social determinants of health, and hospital utilization);

con_before_sdoh contains significant diagnoses negatively associated with male infertility in the social determinants of health sensitivity analysis only.

File Name: Supplementary Data 13

Description: Overlapping diagnoses that are significantly associated with male infertility across all six analyses at UC and Stanford.

mi_b4_both contains significant diagnoses positively associated with male infertility across all six logistic regression analyses before the 6-month cutoff;

mi_aft_both contains significant diagnoses positively associated with male infertility across all six logistic regression analyses after the 6-month cutoff;

con_b4_both contains significant diagnoses negatively associated with male infertility across all six logistic regression analyses before the 6-month cutoff;

legend contains column descriptions.

File Name: Supplementary Data 14

Description: Logistic regression results for each diagnosis first obtained after the 6-month cutoff at UC. Note, patient counts less than or equal to 10 are set to 10.

after_primary contains results for the primary analysis;

after_sdoh contains results for the social determinants of health sensitivity analysis;

after_hosp contains results for the hospital utilization sensitivity analysis;

legend contains column descriptions.

File Name: Supplementary Data 15

Description: Logistic regression results for each diagnosis first obtained after the 6-month cutoff at Stanford. Note, patient counts less than or equal to 10 are set to 10.

after_primary contains results for the primary analysis;

after_sdoh contains results for the social determinants of health sensitivity analysis;

after_hosp contains results for the hospital utilization sensitivity analysis;

legend contains column descriptions.

File Name: Supplementary Data 16

Description: Overlapping diagnoses that are significantly associated with male infertility across analyses at UC for diagnoses first obtained after the 6-month cutoff. phenotype corresponds to diagnosis; phecode category is the disease category of the phenotype (i.e., diagnosis).

mi_after_primary contains significant diagnoses positively associated with male infertility in the primary analysis only;

mi_after_primary_sdoh contains significant diagnoses positively associated with male infertility in the primary analysis and social determinants of health sensitivity analysis;

mi_after_primary_sdoh_hosp contains significant diagnoses positively associated with male infertility in all three analyses (primary, social determinants of health, and hospital utilization);

mi_after_sdoh contains significant diagnoses positively associated with male infertility in the social determinants of health sensitivity analysis only;

con_after_hosp contains significant diagnoses negatively associated with male infertility in the hospital utilization sensitivity analysis only;

con_after_primary_hosp contains significant diagnoses negatively associated with male infertility in the primary analysis and hospital utilization sensitivity analysis;

con_after_primary_sdoh_hosp contains significant diagnoses negatively associated with male infertility in all three analyses (primary, social determinants of health, and hospital utilization).

File Name: Supplementary Data 17

Description: Overlapping diagnoses that are significantly associated with male infertility across analyses at Stanford for diagnoses first obtained after the 6-month cutoff. phenotype corresponds to diagnosis; phecode category is the disease category of the phenotype (i.e., diagnosis).

mi_after_primary contains significant diagnoses positively associated with male infertility in the primary analysis only;

mi_after_primary_sdoh contains significant diagnoses positively associated with male infertility in the primary analysis and social determinants of health sensitivity analysis;

mi_after_primary_sdoh_hosp contains significant diagnoses positively associated with male infertility in all three analyses (primary, social determinants of health, and hospital utilization);

mi_after_sdoh contains significant diagnoses positively associated with male infertility in the social determinants of health sensitivity analysis only;

con_after_hosp contains significant diagnoses negatively associated with male infertility in the hospital utilization sensitivity analysis only.

File Name: Supplementary Data 18

Description: Number of patients lost for each time cutoff for the after 6-month analyses with fixed follow-up times at UC and Stanford.

File Name: Supplementary Data 19

Description: Logistic regression results for each diagnosis first obtained after the 6-month cutoff at UC and within 12, 24, 36, 48, or 60 months of follow-up. Note, patient counts less than or equal to 10 are set to 10.

x_primary contains results for the primary analysis;

x_sdoh contains results for the social determinants of health sensitivity analysis;

x_hosp contains results for the hospital utilization sensitivity analysis;

legend contains column descriptions.

x = 12, 24, 36, 48, or 60. Denotes number of months of follow-up.

File Name: Supplementary Data 20

Description: Logistic regression results for each diagnosis first obtained after the 6-month cutoff at Stanford and within 12, 24, 36, 48, or 60 months of follow-up. Note, patient counts less than or equal to 10 are set to 10.

x_primary contains results for the primary analysis;

x_sdoh contains results for the social determinants of health sensitivity analysis*;

x_hosp contains results for the hospital utilization sensitivity analysis;

legend contains column descriptions.

*48_sdoh does not include the phenotype Late effects of cerebrovascular disease due to singular matrix occurrence.

x = 12, 24, 36, 48, or 60. Denotes number of months of follow-up.

File Name: Supplementary Data 21

Description: Results from Cox proportional hazards models for the 13 diagnoses found to be significantly associated with male infertility across the primary, social determinants of health, and hospital utilization logistic regression analyses at UC.

cox_primary contains results for the primary Cox proportional hazards models;

cox_sdoh contains results for the social determinants of health Cox proportional hazards models;

cox_hosp contains results for the hospital utilization Cox proportional hazards models;

legend contains column descriptions.

File Name: Supplementary Data 22

Description: Overlapping diagnoses that are significantly associated with male infertility for the primary analyses at UC for diagnoses first obtained before or after the 6-month cutoff. phenotype corresponds to diagnosis; phecode category is the disease category of the phenotype (i.e., diagnosis).

mi_b4_vs_aft_b4 contains significant diagnoses positively associated with male infertility in the before 6-month cutoff primary analysis only;

mi_b4_vs_aft_aft contains significant diagnoses positively associated with male infertility in the after 6-month cutoff primary analysis only;

mi_b4_vs_aft_b4_aft contains significant diagnoses positively associated with male infertility in both primary analyses.

File Name: Supplementary Data 23

Description: Overlapping diagnoses that are significantly associated with male infertility for the primary analyses at Stanford for diagnoses first obtained before or after the 6-month cutoff. phenotype corresponds to diagnosis; phecode category is the disease category of the phenotype (i.e., diagnosis).

mi_b4_vs_aft_b4 contains significant diagnoses positively associated with male infertility in the before 6-month cutoff primary analysis only;

mi_b4_vs_aft_aft contains significant diagnoses positively associated with male infertility in the after 6-month cutoff primary analysis only;

mi_b4_vs_aft_b4_aft contains significant diagnoses positively associated with male infertility in both primary analyses.
