# Supplementary material for: Leveraging electronic health records from two hospital systems identifies male infertility-associated comorbidities across time
Source: Commun Med (Lond). 2025 Sep 1;5:380. doi: 10.1038/s43856-025-01071-7 (PMC12402078; doi:10.1038/s43856-025-01071-7)
Supplement: Supplementary file 27 — Reporting Summary [file 43856_2025_1071_MOESM27_ESM.pdf]

nature portfolio

Corresponding author(s): Dr. Marina Sirota

Last updated by author(s): Jul 18, 2025

Reporting Summary

Nature Portfolio wishes to improve the reproducibility of the work that we publish. This form provides structure for consistency and transparency in reporting. For further information on Nature Portfolio policies, see our Editorial Policies and the Editorial Policy Checklist.

Statistics

For all statistical analyses, confirm that the following items are present in the figure legend, table legend, main text, or Methods section.

n/a	Confirmed	
☐	☒	The exact sample size (*n*) for each experimental group/condition, given as a discrete number and unit of measurement
☒	☐	A statement on whether measurements were taken from distinct samples or whether the same sample was measured repeatedly
☐	☒	The statistical test(s) used AND whether they are one- or two-sided<br>*Only common tests should be described solely by name; describe more complex techniques in the Methods section.*
☐	☒	A description of all covariates tested
☐	☒	A description of any assumptions or corrections, such as tests of normality and adjustment for multiple comparisons
☐	☒	A full description of the statistical parameters including central tendency (e.g. means) or other basic estimates (e.g. regression coefficient) AND variation (e.g. standard deviation) or associated estimates of uncertainty (e.g. confidence intervals)
☐	☒	For null hypothesis testing, the test statistic (e.g. *F*, *t*, *r*) with confidence intervals, effect sizes, degrees of freedom and *P* value noted<br>*Give P values as exact values whenever suitable.*
☒	☐	For Bayesian analysis, information on the choice of priors and Markov chain Monte Carlo settings
☒	☐	For hierarchical and complex designs, identification of the appropriate level for tests and full reporting of outcomes
☐	☒	Estimates of effect sizes (e.g. Cohen's *d*, Pearson's *r*), indicating how they were calculated

Our web collection on statistics for biologists contains articles on many of the points above.

Software and code

Policy information about availability of computer code

Data collection	No new data was collected for this study. No software was used for data collection.
Data analysis	See Methods

For manuscripts utilizing custom algorithms or software that are central to the research but not yet described in published literature, software must be made available to editors and reviewers. We strongly encourage code deposition in a community repository (e.g. GitHub). See the Nature Portfolio guidelines for submitting code & software for further information.

Data

Policy information about availability of data

All manuscripts must include a data availability statement. This statement should provide the following information, where applicable:

- Accession codes, unique identifiers, or web links for publicly available datasets
- A description of any restrictions on data availability
- For clinical datasets or third party data, please ensure that the statement adheres to our policy

UCDDP is only available to UC researchers who have completed analyses in their respective UC first and have provided justification for scaling their analyses across UC health centers. Details on how to access UCDDP for UC researchers can be found in the University of California Health Center for Data-driven Insights and Innovation website (https://www.ucop.edu/uc-health/departments/center-for-data-driven-insights-and-innovations-cdi2.html). Stanford-affiliated researchers may request access to Stanford's EHR data through the STAnford medicine Research data Repository (STARR) (https://starr.stanford.edu/data-models/omop).

Human research participants

Policy information about studies involving human research participants and Sex and Gender in Research.

Reporting on sex and gender	We focused our study on patients identified as being of male biological sex. These patients were identified using the OMOP field gender_concept_id = MALE. While we understand that biological sex and gender are distinct, in OMOP, gender_concept_id refers to biological sex (https://www.ohdsi.org/web/wiki/doku.php?id=documentation:vocabulary:gender).
Population characteristics	Study participants were identified from the UCDDP; these patients received care at either UCD, UCSF, UCI, UCLA, or UCSD. Medical centers 1-5 in the study correspond to one of these UC health centers. For validation, study participants were identified from the de-identified STAnford medicine Research data Repository (STARR) OMOP electronic health record database. All study participants were identified as being of male biological sex. Demographic characteristics are shown in Table 1.
Recruitment	No recruitment was performed for this study.
Ethics oversight	This study has been approved by the Institutional Review Boards of the University of California, San Francisco (#17-22929) and Stanford University (#39225). Analysis of UC-wide de-identified EHR data via the UC Data Discovery Portal was performed by UCSF employees under approval by the UC Health IRBs for research use. Since only de-identified data was analyzed, written informed consent was waived by all UC institutions.

Note that full information on the approval of the study protocol must also be provided in the manuscript.

Field-specific reporting

Please select the one below that is the best fit for your research. If you are not sure, read the appropriate sections before making your selection.

☒ Life sciences ☐ Behavioural & social sciences ☐ Ecological, evolutionary & environmental sciences

For a reference copy of the document with all sections, see nature.com/documents/nr-reporting-summary-flat.pdf

Life sciences study design

All studies must disclose on these points even when the disclosure is negative.

Sample size	No sample size calculation was performed prior to the study. Sample size was determined by the number of patients who either have a male infertility diagnosis or a vasectomy procedure in UCDDP (n=14,884 patients) and Stanford (n=8,015 patients) EHR databases.
Data exclusions	Data from patients not identified as being of male biological sex were excluded due to the study scope encompassing male infertility specifically.
Replication	Not applicable, this study did not acquire new data. Replication of analyses from UCDDP were performed at Stanford.
Randomization	Not applicable, this study did not acquire new data.
Blinding	Not applicable, this study did not acquire new data.

Reporting for specific materials, systems and methods

We require information from authors about some types of materials, experimental systems and methods used in many studies. Here, indicate whether each material, system or method listed is relevant to your study. If you are not sure if a list item applies to your research, read the appropriate section before selecting a response.

Materials & experimental systems

n/a	Involved in the study	
☒	☐	Antibodies
☒	☐	Eukaryotic cell lines
☒	☐	Palaeontology and archaeology
☒	☐	Animals and other organisms
☒	☐	Clinical data
☒	☐	Dual use research of concern

Methods

n/a	Involved in the study	
☒	☐	ChIP-seq
☒	☐	Flow cytometry
☒	☐	MRI-based neuroimaging
